# Does effective population size affect rates of molecular evolution: Mitochondrial data for host/parasite species pairs in bees suggests not

**DOI:** 10.1002/ece3.8562

**Published:** 2022-02-07

**Authors:** Nahid Shokri Bousjein, Simon M. Tierney, Michael G. Gardner, Michael P. Schwarz

**Affiliations:** ^1^ 1065 College of Science and Engineering Flinders University Adelaide South Australia Australia; ^2^ 145440 Faculty of Biological Sciences Kharazmi University Tehran Iran; ^3^ Hawkesbury Institute for the Environment Western Sydney University Penrith New South Wales Australia; ^4^ Evolutionary Biology Unit South Australian Museum North Terrace Adelaide South Australia Australia

**Keywords:** neutral theory, obligate social parasitizes, relaxed selection, slightly deleterious mutations

## Abstract

Adaptive evolutionary theory argues that organisms with larger effective population size (*N*_e_) should have higher rates of adaptive evolution and therefore greater capacity to win evolutionary arm races. However, in some certain cases, species with much smaller *N*_e_ may be able to survive besides their opponents for an extensive evolutionary time. Neutral theory predicts that accelerated rates of molecular evolution in organisms with exceedingly small *N*_e_ are due to the effects of genetic drift and fixation of slightly deleterious mutations. We test this prediction in two obligate social parasite species and their respective host species from the bee tribe Allodapini. The parasites (genus *Inquilina*) have been locked into tight coevolutionary arm races with their exclusive hosts (genus *Exoneura*) for ~15 million years, even though *Inquilina* exhibit *N*_e_ that are an order of magnitude smaller than their host. In this study, we compared rates of molecular evolution between host and parasite using nonsynonymous to synonymous substitution rate ratios (d_N_/d_S_) of eleven mitochondrial protein‐coding genes sequenced from transcriptomes. Tests of selection on mitochondrial genes indicated no significant differences between host and parasite d_N_/d_S,_ with evidence for purifying selection acting on all mitochondrial genes of host and parasite species. Several potential factors which could weaken the inverse relationship between *N*_e_ and rate of molecular evolution are discussed.

## Graphical abstract

This study investigates a critical hypothesis in the field of evolutionary biology – whether rates of molecular evolution are higher in organisms with much smaller effective population sizes. To examine this issue, we compared rates of mitochondrial genes evolution between Australian host and parasite bee species when their effective population sizes differ markedly. Surprisingly, our analyses did not support predictions that rates of mitochondrial evolution are higher in parasite species compared to their hosts.

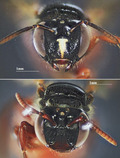

## 1 INTRODUCTION

There has been very substantial and sustained interest in how the evolutionary rates of species vary with selection pressure and effective population size (*N*_e_): a function of the number of individuals that contribute alleles to each generation (Kimura & Ohta, 1971; Woolfit, 2009; Woolfit & Bromham, 2003). One particular aspect of this interest has focused on evolutionary “arms races” between species that are locked into competition, such as hosts and their parasites, but which differ in their capacities for evolutionary rates (Kimura & Ohta, 1971; Lanfear et al., 2014; Woolfit, 2009). Very generally, evolutionary rates are influenced by selection and genetic drift. Both selection and drift can be strongly influenced by *N*_e_. Larger populations provide more opportunities for mutations to enter each generation and hence be subjected to selection, and this can equate to more favorable mutations being available for selection to promote, leading to high rates of molecular evolution. On the other hand, smaller populations are expected to be subjected to higher rates of genetic drift which should also lead to relatively high evolutionary rates for neutral mutations and even deleterious mutations. These two different considerations of population size lead to specific predictions (Woolfit, 2009; Woolfit & Bromham, 2003), which we now briefly discuss.

For populations or species with relatively small *N*_e_, Ohta’s (1973, 1992) nearly neutral theory predicts that most nonsynonymous nucleotide substitutions will fall into a “nearly neutral” state – wherein extremely deleterious mutations should still be removed by selection and highly favorable mutations should be rare. Under this scenario, the nonsynonymous substitution rate (d_N_) for “slightly” deleterious mutations increases because of enhanced genetic drift while d_N_ for the slightly advantageous mutations decreases because small population size will lower the number of favorable mutations entering a gene pool; and also, slightly advantageous mutations are likely to be lost due to the lowered efficacy of positive selection. Nevertheless, given that slightly detrimental mutations comprise a substantial proportion of mutations in lineages with small *N*_e_, previous studies (Eyre‐Walker et al., 2002; James et al., 2016; Woolfit, 2009) have suggested that the ratio of non‐synonymous to synonymous substitution rates (referred to as ω or rate of molecular evolution) should be greater in lineages with relatively small *N*_e_ (Ohta, 1992).

The ratio ω (d_N_/d_S_) can provide an indication of the mode and strength of selection acting on protein‐coding genes (Nielsen, 2005; Yang & Bielawski, 2000), where ω = 1 signifies neutral evolution, ω ~ 0 is suggestive of strong selective constraints, ω < 1 indicates purifying selection and ω > 1 indicates strong positive selection. Empirical measurements of ω can therefore provide insights into the history of selection relative to population sizes of particular lineages and focal genes (Wagner, 2002).

### 1.1 Practical consequences of varying effective population sizes

The above issues regarding ω and *N*_e_ can potentially become very problematic for species that are locked into co‐evolutionary arms races with other species that have much larger effective population sizes, and consequently potentially greater rates of adaptive evolution (Shokri Bousjein et al., 2016, 2017). Such a situation could arise in obligate parasite‐host associations (species dyads) if parasites have much smaller *N*_e_ than their hosts, where we might expect hosts to have higher rates of adaptive evolution and parasites to accumulate slightly deleterious mutations more rapidly, as suggested for some species of socially parasitic inquiline bees (Shokri Bousjein et al., 2016).

Several studies have compared rates of molecular evolution in organisms assumed to have different effective population sizes, but they have not produced consistent results. Spradling et al. (2001) compared the rate of cytochrome *b* evolution in 21 rodent species and found an inverse relationship between effective population size and the rate of molecular evolution. Johnson and Seger (2001) showed an increase in evolutionary rates of island avian species, which are supposed to have smaller effective population sizes compared to those occurring on the mainland. Furthermore, Woolfit and Bromham (2003) argued that long‐term reductions in population sizes of endosymbiotic microorganisms compared to their free‐living relatives caused an increase in the nonsynonymous substitution rates of the 16S rRNA gene. Bromham and Leys (2005) conducted a comparative analysis on social parasites of bees, wasps, and ants, using concatenated mtDNA, nuclear and ribosomal genes, and found that social parasites tend to have faster rates of nonsynonymous substitution than their social hosts, which they attributed to the effect of smaller effective population of parasites on the rate of molecular evolution. In contrast, Erler et al. (2014) found similar rates of evolution among almost all defense‐related genes (antimicrobial peptide genes) when comparing host and socially parasitic bumblebees. A study by Helbing and Lattorff (2016) revealed that three antiviral siRNA genes evolved faster in host bumblebees compared to their respective parasitic species. Furthermore, Fouks and Lattorff (2016) discovered similar protein evolutionary rates for nuclear gene *EF‐1α* between social *Bombus terrestris* and its parasitic species *B. vestalis*.

To further examine this issue, we compared rates of mitochondrial evolution using two allodapine bee host species and their obligate social parasite bee species, otherwise known as allodapine inquilines. These inquilines very rarely infest more than 5% of host colonies (Smith et al., 2013; Smith & Schwarz, 2006a, 2009). They are locked in tight co‐evolutionary arms races with their host because they are obligate parasites and host‐specific (Smith et al., 2013; Smith & Schwarz, 2009). These parasitic species spend their almost entire life cycle within the nest of the host species and have extreme adaptations to social parasitism, including strongly reduced mouth parts and pollen‐collecting scopae. Given these morphological variations, they are completely dependent on their host's colony for brood rearing (Michener, 1965, 1970b, 1971a, 1975, 1983). Allodapine host and parasite clades are mostly sibling lineages (Smith et al., 2013) and their life‐history traits such as body size and generation times are similar (Michener, 1965; Smith & Schwarz, 2006a, 2006b), making this group a good model system for examining rates of evolution in both hosts and parasites.

The Australian inquiline genus *Inquilina* Michener forms the sister clade to its host genus *Exoneura* Smith (Chenoweth & Schwarz, 2011; Smith et al., 2013). A previous study estimated the relative *N*_e_ of *Exoneura* and its parasite *Inquilina* based on the mean number of reproductive host and parasite females per nest and found that *N*_e_ of parasite species is at least an order of magnitude lower than its host (Shokri Bousjein et al., 2016). Despite this, previous phylogenetic analyses of allodapine parasites by Smith et al. (2013) showed that they have been able to persist for long periods of evolutionary time (about 15 million years ago from their initial divergence) and are presumed to have followed their hosts through multiple speciation events.

In this study, we examine whether the much smaller effective population sizes of allodapine parasite species in the genus *Inquilina* give rise to faster rates of molecular evolution (dN/dS) compared to their *Exoneura* host species; as theoretically predicted (Woolfit, 2009; Woolfit & Bromham, 2003). We target mitochondrial genes to examine this hypothesis because mitochondrial DNA is highly variable in many animal species because of its elevated mutation rate, which stands as ideal marker for the study of evolutionary events among relatively close phylogenetic species, such as allodapine host‐parasite dyads (Galtier et al., 2009).

Given that we have focused on the rate of molecular evolution (ω) as measures of the efficiency of selection, our a priori expectation is that selection should be relaxed on mitochondrial genes of parasite species compared to their hosts because of the predicted enhanced effects of genetic drift on species with small *N*_e_ (Ohta, 1973; Weber & Diggins, 1990; Woolfit, 2009). In other words, the ratio of non‐synonymous (slightly deleterious substitutions) to synonymous substitution (neutral substitutions) rates of mtDNA genes (referred to d_N_/d_S_ or ω or rate of molecular evolution) should be greater in *inquilina* species with much smaller *N*_e_ compared to *Exoneura* species.

## 2 MATERIALS AND METHODS

### 2.1 Sampling methods

Our study focused on *Inquilina schwarzi* Michener, 1983 and *Inquilina excavata* Cockerell 1922, which infest colonies of the semisocial allodapine bees *Exoneura robusta* Cockerell 1922 and *E. angophorae* Cockerell 1912 respectively (Smith & Schwarz, 2009).

**FIGURE 1:**
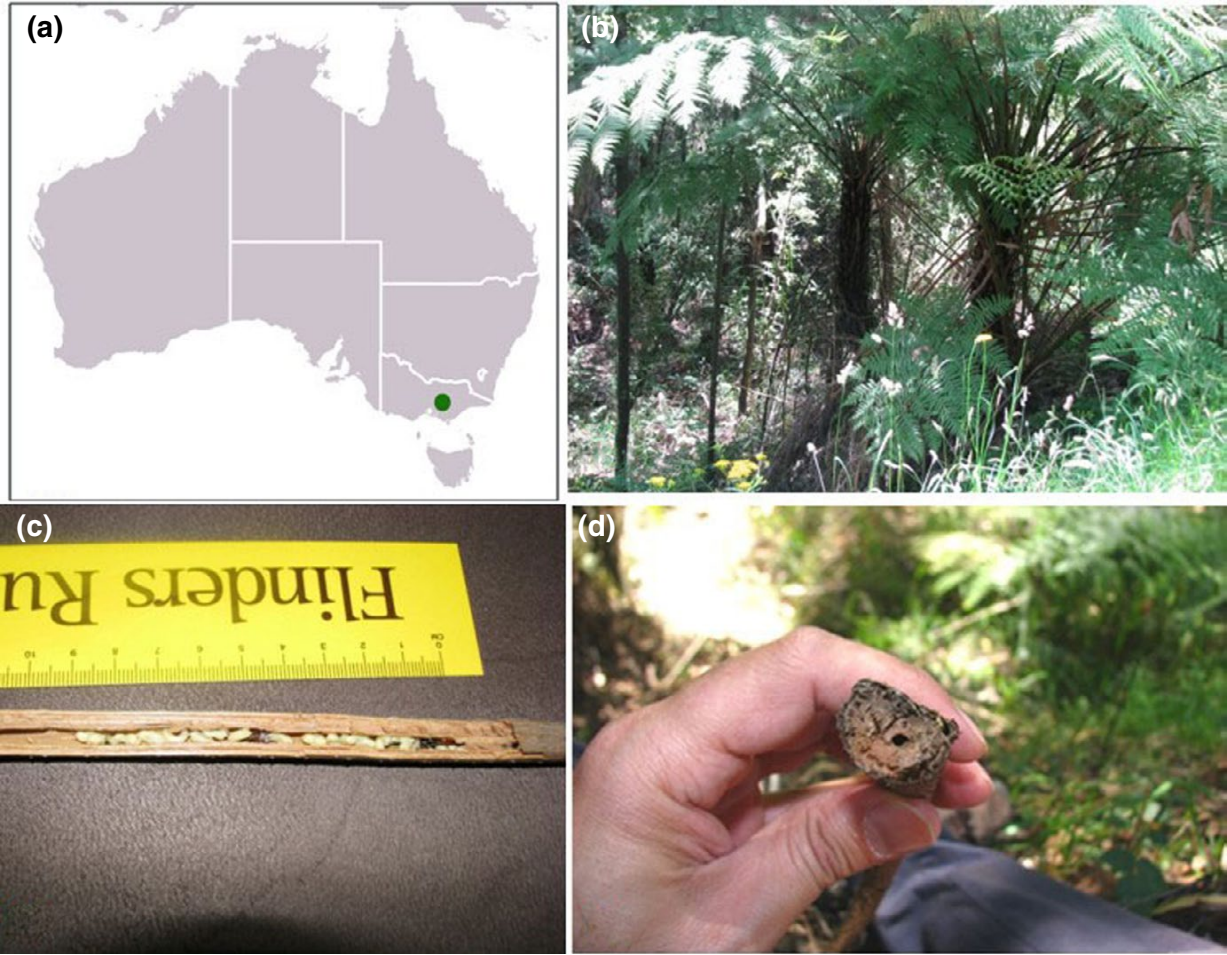
(a) Green dot indicates collection locality of *Exoneura* and *Inquilina* in this study (This image was adapted from https://commons.wikimedia.org/wiki/File:Australia_location_map_grey.svg#file); (b) fronds of the tree fern *Cyathea australis* containing *Exoneura* nests; (c) view of the basal end of a dead tree fern frond, *Cyathea australis*, showing the entrance of an *Exoneura* nest; (d) longitudinal bisection of nest containing adult females and pupae

Sampling was undertaken in December 2013, from the Gembrook region in the Dandenong Ranges of Victoria, Australia. Nests containing host and parasite species were collected from dead and fallen fronds of the tree fern, *Cyathea australis*, early in the morning when bees were not active (Figure 1). All collected nests were immediately stored in insulated boxes on ice and transported back to Flinders University for nest dissection. Ethics approval was not required for this study.

### 2.2 RNA preparation and high‐throughput sequencing

Specimens were snap frozen on dry ice and the head and metasoma of each species were dissected on dry ice in the laboratory and immediately preserved in RNAlater^®^ (Weber et al., 2010) to prevent RNA degradation. In order to replicate sampling procedures, head and metasomal tissues were analyzed separately, and we pooled tissues from 2–3 individuals per species. Total RNA from pooled tissues was extracted using RNeasy^®^ PlusMicro Kit (Qiagen). SMARTer^TM^ cDNA Synthesis Kit and Advantage^®^2 PCR Kit (Clontech Laboratories, Inc) were used to synthesize double‐stranded complementary DNA and PCR‐amplify products. PCR cycle optimization was assessed on gel and products were purified using Ultraclean^®^ PCR Clean up kit (MO BIO Laboratories, Inc). DNA library preparation and sequencing were outsourced to Australian Genome Research Facility (AGRF) for sequencing on an Illumina HiSeq platform, which generated 100 bp paired‐end reads.

### 2.3 Bioinformatics

#### 2.3.1 Quality control, assembly, and gene orthology

Raw sequence data was quality controlled using FASTQC (Babraham Institute) and trimmed using CUTADAPT (Martin, 2011) to remove Nextra adapters, SMARTER PCR Primers, reads containing suspected poly‐Adenine and poly‐Thymine tails, low quality reads (phred scores <30), and sequences shorter than 25 bp. The Trinity platform was used for *de novo* assembly of transcript sequences using a default k‐mer of 25 (Haas et al., 2013; Tierney et al., 2015). The quality of assembled contigs was then assessed by mapping them back to raw reads using the BOWTIE 2 aligner and the quantity of proper‐paired reads was calculated. Read coverage statistics were also retrieved using BEDTOOLS (v.2.22.0). We used two approaches to determine and verify orthologous genes, sequence similarity and phylogenetic reconstruction (Tekaia, 2016; Tierney et al., 2015).

##### Sequence similarity inference

We matched assembled transcripts using the BLASTX algorithm against seven NCBI reference species (mitochondrial protein‐coding genes), with a 10^−6^ E‐value cut‐off. Reference species used in this study included the African honey bee *Apis* mellifera *scutellata* Linnaeus 1758 (Hymenoptera: Apidae); red dwarf honey bee *Apis florea* Fabricius 1787 (Hymenoptera: Apidae); Guaraipo bee *Melipona bicolor* Lepeletier 1836 (Hymenoptera: Apidae); Urussu bee *Melipona scutellaris* Latreille 1811 (Hymenoptera: Apidae); bumble bee *Bombus ignitus* Smith 1869 (Hymenoptera: Apidae); plasterer bee *Colletes gigas* Cockerell 1918 (Hymenoptera: Colletidae); and chalk yellow face bee *Hylaeus dilatatus* Kirby 1802 (Hymenoptera: Colletidae; Table S1). We then ran two reciprocal best similarity hit methods (tBLASTn and BLASTn), with a 10^−6^ E‐value threshold to verify putative orthologs. We considered stringent criteria to call reciprocal best hits as orthologs if we found: alignment length ≥50%; protein identity ≥30%; and nucleotide similarity ≥50% (Tommaso et al., 2011). However, a few best hit contigs didn't match to any of the reference species mtDNA genes due to their short length. We therefore performed BLAST2BLASTN alignment between best‐hit contigs that were perfectly matched to reference species mtDNA genes (subject sequence) and un‐matched best‐hit contigs (query sequence); as per Tierney et al. (2015).

##### Phylogenetic reconstructions

For each species, a consensus sequence was generated in Geneious v.9.1.4 (Kearse et al., 2012) from best hit contigs of head and metasomal tissue using pairwise alignment, Geneious algorithm, setting global alignments with free end gaps for alignment type, 65% similarity for cost matrix, 9 for gap open penalty, 3 for gap extension penalty and allowing determine sequence directions automatically. In the case of heterozygote sites between best hit contigs of head and metasomal tissues, the IUPAC ambiguity codes were used. Multiple sequence alignments of consensus sequences and reference orthologous genes were created using Translation alignment and MAFFT/Geneious algorithms. Amino acid sequences were translated using the standard invertebrate mitochondria code and examined visually for unusual features.

A matrix of nucleotide alignments was then analyzed using a Bayesian inference method implemented in MrBayes v.3.2 (Huelsenbeck & Ronquist, 2001). Two different approaches were used to reconstruct Bayesian gene trees. Gene trees for each mtDNA gene were reconstructed using both host/parasite focal species and the reference species. We also constructed a phylogenetic tree by concatenating all mtDNA genes.

For MCMC analyses of each individual mtDNA genes, we partitioned nucleotide sequences by codon positions and applied a GTR + I + Γ nucleotide substitution model (the least restrictive model was chosen to avoid potential errors related to incorrect a priori model selection) for each codon partition. Nucleotide sequences were partitioned by genes for MCMC analyses of concatenated mtDNA genes. Analyses were run two times, with each run comprising 10 million generations, using three heated chains and one cold chain and with variable rate permitted, and sampling every 1000th iteration. Likelihood plots and standard deviation of split frequencies (below 0.01) were used to verify stationarity distribution and length of run. Parameter trace files of each run were also examined in TRACER v.1.6 (Rambaut et al., 2014) where the first 10% of trees were discarded as burn‐in FigTree v1.3.1 (Rambaut & Drummond, 2010) was then used to visualize the Bayesian Inference (BI) phylogenetic tree and *Hylaeus dilatatus* and *Colletes gigas* were used as outgroups to root the tree.

### 2.4 Detection of selection pressure

Given that purifying selection is the most prevalent form of selection in essential genes (e.g., mtDNA genes) (Comas et al., 2010; Jordan et al., 2002; Monteiro et al., 2010), we assumed this was also true in our focal bees mtDNA genes unless we found signals of non‐negative selection. For this, we used a Branch‐site Unrestricted Statistical Test for Episodic Diversification (BUSTED) implemented in HyPhy v.2.5 (Kosakovsky Pond et al., 2005) to allocate what proportions of the mtDNA sites select positively (ω > 1). Besides this, BUSTED assigns what percentages of the sites evolve with negative selection (ω < 1).

BUSTED splits branches into foreground and background partitions and fits a codon model with three rate categories as ω_1_ ≤ ω_2_ ≤ 1 ≤ ω_3_ for each partition. It then estimates the proportion of sites per partition belonging to each ω class. This model is referred to as unconstrained model or alternative model. If the unconstrained model shows evidence of positive selection (ω_3_ > 1) and proportion of sites assigned to that category is non‐zero, BUSTED fits the constrained model (null model) where positive selection on the foreground branches is disallowed by constraining ω_3_ = 1. A likelihood ratio test is then used to compare the unconstrained model with the constrained one. If the null hypothesis is rejected, it indicates that at least one site, at least some of the time, is under positive selection on the foreground branches.

### 2.5 Comparison of mitochondrial evolutionary rate

Hyphy v.2.5 was utilized to compare rates of molecular evolution of mtDNA genes between each host and its associated parasite using a portion of inferred concatenated Bayesian tree (containing two focal hosts and their respective parasite species) and the likelihood function created by Hyphy.

Evolutionary comparisons were carried out at two levels:

1. We first estimated branch‐by‐branch variation in rates where all codons of each branch are supposed to have equal rate of evolution (Kosakovsky Pond et al., 2005). These analyses were carried out by fitting the global model (this model posits that ω does not vary from branch to branch) as a null hypothesis (H_0_), with the local model (which allows a separate ω in every branch of the tree) as the alternative hypothesis (H_A_). Likelihood ratio tests (LRT) were then used to explore evidence of branch‐by‐branch rate heterogeneity if H_0_ was rejected.
2. We then compared the rate of molecular evolution when codons within a branch were assumed to have different evolutionary rates. A hypothesis testing framework RELAX 3 (a part of the Hyphy software package) (Wertheim et al., 2015) was used to compare rates of molecular evolution between host and parasite species using the efficacy of selection and quantifying the ω ratio.
3. RELAX partitions branches as two subsets including ‘'test'’ and "reference'’ branches. RELAX then estimates a separate discrete distribution of ω for each of these branch classes (ω_T_ and ω_R_ for test and reference branches respectively). A selection intensity parameter K (where k ≥ 0) is calculated to test for relation/intensification of selection on test branches relative to reference branches. Accordingly, each ω component of the test branches (ω_T_) is obtained by raising the corresponding component of the reference branch (ω_R_) to the power of k: ω_T_ = ω_R_^K^. RELAX then conducts a likelihood ratio test (LRT) by comparing the null model in which k is constrained to 1 (consequently the same ω distribution on test and reference branches or ω_T_ = ω_R_) to an alternative model in which k is allowed to differ between reference and test branches. A statistically significant result of k > 1 means that selection has been intensified along test branches and a significant result of k<1 indicates that selection has been relaxed along test branches.

We tried four different treatments on host and parasite branches for RELAX analyses as follows. Under the first treatment, the combined parasite clade was compared against the combined host clade where parasite clade was identified as test group and host clade as reference one (electronic supplementary material, Figure S2, Treatment 1). For the second treatment, the rate of evolution was compared between the common ancestral lineages for each of the host and parasite clades. For this, stem lineage of host and parasite clades were considered as reference and test branches respectively (electronic supplementary material, Figure S2, Treatment 2). Each parasite (test branch) was also compared with its own host (reference branch) for the third and fourth treatments (electronic supplementary material, Figure S2, Treatments 3 and 4). These treatments were first examined on individual gene because the rate of evolution is not equal between different mtDNA genes (Rand, 2001). However, given that mtDNA genes are completely linked and therefore, are not independent replicates of evolution (Mitterboeck & Adamowicz, 2013), we concatenated all mitochondrial genes into a single alignment and repeated all treatments for concatenated mtDNA genes (Figure S2).

## 3 RESULTS

### 3.1 Transcriptome assembly statistics

In total, Illumina sequencing is generated between 27.3 and 37.7 million paired‐end reads, of which 16.6–29.2 million remained after quality trimming. Total assembled transcripts varied from 28,372–82,102 per species, and contig N50 ranged from 552–1526 bp. Overall, 30.4–54.3 million reads in the assembled files were aligned to the post‐trimming transcripts and 52.83%–77.62% of reads were determined as proper pairs. Detailed summary statistics on reads and assembly can be found in Table S2.

### 3.2 Orthology

BLASTX searches of the assembled contigs against reference species recovered 10–11 protein‐coding mtDNA genes of hosts and parasites species. These genes comprised COI, CO2, CO3, Cyt‐b, ND‐1, ND‐3, ND‐4, ND‐4L, ND‐5, ATP6, and ATP8. Of these, ND‐3 was the only one not recovered in *I. schwarzi*. In total, Illumina sequencing of hosts and parasites transcripts covered 77%–85% of the reference species mitochondrial protein‐coding genes. Both reciprocal best hit methods showed that the majority of suggested contigs derived from BLASTX search met the defined criteria (alignment length ≥50%; protein identity ≥30%; and nucleotide similarity ≥50%). ND‐4L of both host species and *I. schwarzi* was the only gene which failed to meet those standards. We therefore undertook BLAST2BLASTN alignment using best‐hit contigs of *I. excavata* to verify orthology of ND‐4L best hit contigs from each species. Information concerning the length and depth coverage of identified mtDNA genes of focal host and parasite species is shown in Table 1 and supplementary material, S3 respectively.

**TABLE 1:** Characteristics of the mitochondrial genes of focal species

Size(bp)/Species	ATP6	ATP8	CO1	CO2	CO3	Cyt‐b
*E. robusta*	684	156	1538	624	762	1135
	OL829924	OM022056	OM022054	OM022062	OM022065	OM022069
*E. angophorae*	684	156	1537	648	736	1090
	OL829923	OM022059	OM022052	OM022063	OM022064	OM022068
*I. excavata*	684	156	1538	675	769	1137
	OL829925	OM022057	OM022053	OM022061	OM022066	OM022070
*I. schwarzi*	684	156	1538	675	691	1134
	OL829926	OM022058	OM022055	OM022060	OM022067	OM022071

Size(bp)/Species	ND−1	ND−3	ND−4	ND−4L	ND−5
*E. robusta*	891	354	1286	115	1167
	OM022073	OM022089	OM022077	OM022087	OM022081
*E. angophorae*	909	246	1280	100	919
	OM022074	OM022090	OM022076	OM022086	OM022080
*I. excavata*	572	267	1284	102	1166
	OM022075	OM022088	OM022078	OM022085	OM022082
*I. schwarzi*	912	Not found	1287	111	1086
	OM022072	–	OM022079	OM022084	OM022083

Note

The mtDNA best hit contigs derived from BLASTx which met the criteria of tBLASTn and BLASTn (alignment length ≥50%; protein identity ≥30% and nucleotide similarity ≥50%) are highlighted. ND‐4L best hit contigs of both host and one inquiline species didn't meet those criteria and obtained from BLAST2BLASTN alignment. Genebank accession numbers were provided below the size of each mtDNA genes.

### 3.3 Phylogenetic tree inference

**FIGURE 2:**
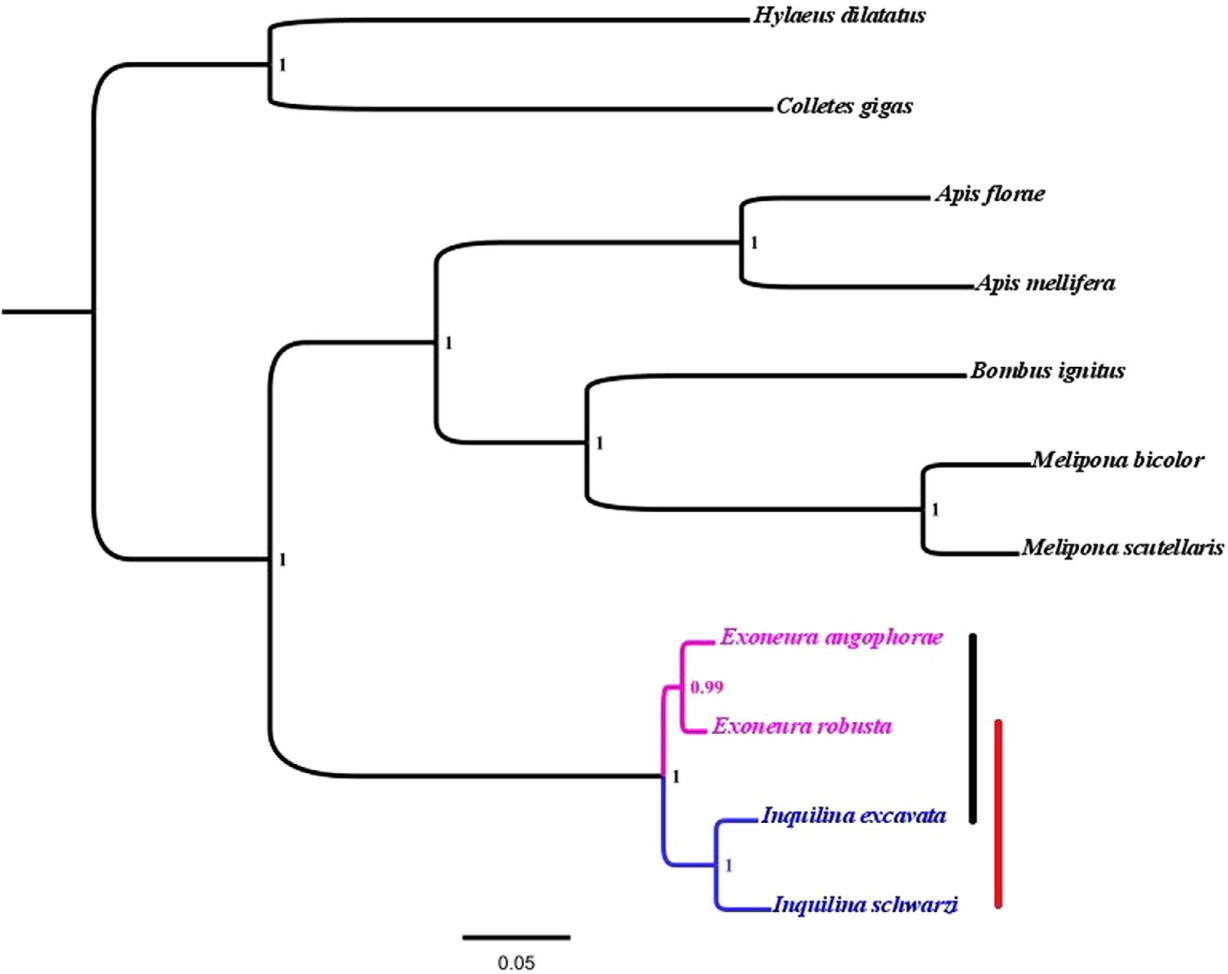
Mitochondrial genes phylogeny. A consensus tree (from MrBayes analyses) derived from 11 concatenated protein‐coding mtDNA genes, with posterior probability node support. Parasites and hosts are indicated by colored branches: pink for hosts and blue for parasites. The lines to the right of the terminal branches link each host to its associated parasite

Gene trees based on individual genes were sometimes poorly supported (Figure S1) which is not unexpected given the short sequences for some genes. We therefore present here a phylogenetic tree of concatenated all mtDNA genes (Figure 2). The concatenated mitochondrial sequences retained for analyses were 8948 bp long. The inferred Bayesian tree (with posterior probability node support) corroborated BLAST methods results. It revealed that all predicted mtDNA genes of hosts and parasites species formed a fully resolved monophyletic clade with maximal posterior probability (PP) support (PP = 1.0).

### 3.4 Test of selection

Although tree wide global rate of selection (Table 3) and all ω values yielded from RELAX analyses (Table 4) were definitely suggestive that all host and parasite mtDNA genes have undergone purifying selection, we didn't take those point estimates of ω as statistically robust measures of selection as both tests are not developed to perform for neutrality test (Kosakovsky Pond et al., 2005; Wertheim et al., 2015). We instead used BUSTED test to examine whether positive selection has a role in evolution of host and parasite mtDNA genes. BUSTED test showed that constrained (null model) is accepted where no statistically significant evidence of positive selection (ω > 1) on any host and parasite mitochondrial genes was found (Table 2). In addition, values of point estimates of ω calculated by BUSTED revealed strong evidence of purifying selection acting on mtDNA genes (reported as ω_1_ and ω_2_ in Table 2).

**TABLE 2:** Branch‐site Unrestricted Statistical Test for Episodic Diversification (BUSTED) summary

mtDNA genes	*Log* L constrained model	*Log* L unconstrained model	AICc constrained model	AICc unconstrained model	BUSTED *p* value	ω_1_	ω_2_
ATP6	−1003.3	−1003.1	2064.5	2066.1	.804	0.00	0.13
ATP8	−216.7	−216.1	498.6	500.1	.540	0.00	0.00
CO1	–	−2232.4	–	4523.6	1.000	0.00	0.36
CO2	–	−996.1	–	2052.1	1.000	0.08	0.08
CO3	–	−1164.6	–	2389.0	1.000	0.11	0.13
Cyt‐b	–	−1677.9	–	3415.1	1.000	0.06	0.07
ND‐1	–	−1181.9	–	2423.3	1.000	0.07	0.08
ND‐3	−414.7	−414.5	874.2	876.1	.831	0.00	0.00
ND‐4	−1813.4	−1813.4	3683.8	3685.8	.950	0.00	0.00
ND‐4L	–	−148.5	–	368.8	1.000	0.03	0.03
ND‐5	–	−1645.5	–	3368.2	1.000	0.03	0.46

Note

Significant positive diversifying selection on each branch was tested using the likelihood ratio test by comparing a constrained model in which positive selection is disallowed on foreground branches and unconstrained model which allows positive selection at a proportion of sites on foreground and background branches. Accordingly, none of the mtDNA genes were found to be under significant positive selection. The statics for constrained model was not produced for mtDNA genes including CO1, CO2, CO3, Cyt‐b, ND‐1, ND‐4L, and ND‐5 because unconstrained model didn't find any evidence of positive selection (ω_3_ > 1) and proportion of sites assigned to that category was also zero. For these genes constrained model is the same as the unconstrained model. The small‐sample Akaike Information Criterion (AICc, Sugiura, 1978) was used to compare the goodness of fit of two models. **ω_1_** and **ω_2_** reported here are calculated under the accepted constrained model.

**TABLE 3:** Tree wide global rate of selection versus local rates

mtDNA genes	Tree wide global rate of selection	Tree wide global rate versus local rates of selection		
		2*Likelihood Ratio (LR)	DF	*p*‐value
ATP6	0.196	2.590	5	.762
ATP8	0.533	1.529	5	.909
CO1	0.0346	9.669	5	.085
CO2	0.127	2.500	5	.776
CO3	0.123	6.544	5	.256
Cyt‐b	0.0786	3.971	5	.553
ND‐1	0.0744	0.580	5	.988
ND‐3	0.256	1.756	3	.624
ND‐4	0.110	8.039	5	.154
ND‐4L	0.044	4.238	5	.515
ND‐5	0.087	8.457	5	.132

Note

Tree wide global rate posits ω does not vary from branch to branch in the tree and provides a crude measure of the overall strength of selection acting on each mtDNA gene. Local model allows a separate ω in every branch of the tree. The likelihood ratio test was used to compare global model (null hypothesis) and local model (alternative hypothesis) on each mtDNA gene and resulted in a very strong (*p* ≥ 0.05 in all cases) support in favor of the global rate. DF = Degrees of freedom.

### 3.5 Comparison of mitochondrial evolutionary rates between host and parasite

#### 3.5.1 Estimating branch‐by‐branch variation in rates

Likelihood ratio tests for each gene resulted in support for global rates, which posits the same ω for all host and parasite branches where all codon positions are assumed to have equal rate of evolution (Table 3).

#### 3.5.2 Estimating codon‐by‐codon variation in rates

We continued our comparisons by allowing variation in rates across all codon positions of each branch based on inferred equal ω among host and parasite branches.

In all treatments, we found no evidence that purifying selection of parasite species is less efficient than host species. In other words, our analyses did not support predictions that rates of molecular evolution in mitochondrial genes are higher in parasite species than their hosts (Table 4).

**TABLE 4:** Test for Relaxed selection of mtDNA genes in parasite species compared with hosts

mtDNA genes	Treatments	Relaxation coefficient (k)	*p* Value	LR	ω test / reference branches	AICc Null model	AICc Alternative model
ATP6	1	1.55	.252	1.31	0.162	2057.99	2058.80
	2	1.04	.959	0.00	0.0001	2074.67	2076.83
	3	1.57	.328	0.95	0.153	2073.02	2074.22
	4	1.00	.978	0.00	0.000	2072.55	2074.72
ATP8	1	1.00	.976	0.00	0.00	489.39	492.02
	2	1.13	.976	0.00	0.00	514.22	517.13
	3	1.00	.979	0.00	0.00	514.22	517.14
	4	1.00	.977	0.00	0.00	513.47	516.39
CO1	1	0.78	.122	2.38	0.0193	4521.09	4520.78
	2	49.75	.486	0.48	0.0329	4531.17	4532.76
	3	1.07	.744	0.11	0.0105	4532.07	4534.04
	4	0.67	.107	2.58	0.0215	4534.02	4533.51
CO2	1	0.75	.307	1.04	0.0893	2047.53	2048.61
	2	1.00	.992	0.00	0.0413	2060.51	2062.68
	3	0.57	.240	1.38	0.0931	2065.60	2066.39
	4	0.96	.885	0.02	0.0918	2061.16	2063.30
CO3	1	0.56	.050	3.81	0.111	2385.22	2383.52
	2	3.85	.528	0.40	0.0001	2396.03	2397.78
	3	1.01	.979	0.00	0.249	2397.65	2399.79
	4	0.61	.251	1.32	0.079	2398.96	2399.79
ND‐1	1	1.18	.519	0.42	0.071	2415.20	2416.87
	2	9.94	.987	0.00	0.063	2433.39	2435.51
	3	0.97	.926	0.01	0.066	2433.40	2435.51
	4	1.49	.473	0.51	0.087	2433.91	2435.51
ND‐3	1	1.00	.979	0.00	0.00	893.77	896.18
	4	1.00	.971	0.00	0.00	898.53	900.97
ND‐4	1	0.50	.116	2.47	0.020	3685.31	3684.91
	2	1.13	.978	0.00	0.070	3692.33	3694.41
	3	0.23	.192	1.70	0.065	3695.26	3695.65
	4	1.02	.976	0.00	0.00	3693.16	3695.24
ND‐4L	1	1.46	.598	0.28	0.070	358.96	361.57
	2	1.13	.999	0.00	1.00	382.55	385.88
	3	0.26	.317	1.00	0.0667	383.55	385.89
	4	1.00	.989	0.00	0.00	382.55	385.88
ND‐5	1	0.04	.136	2.22	0.00	3361.79	3361.64
	2	1.13	.983	0.00	0.00	3372.16	3374.25
	3	0.03	.106	2.61	0.031	3374.85	3374.34
	4	1.16	.662	0.19	0.034	3372.40	3374.30
Concatenated genes	1	1.89	.0944	2.79	0.014	25,441.575	25,440.788
	2	4.680	.329	0.952	0.00	25,442.154	25,443.213
	3	0.902	.436	0.605	0.069	25,443.003	25,444.409
	4	1.050	.845	0.038	0.0162	25,444.114	25,446.088

Note

The RELAX test was used to examine whether selection is relaxed or intensified on a subset of test branches compared with a subset of reference branches in a predefined tree. The relaxation coefficient, k, is used to estimate selection intensity. In the null model, the selection intensity is constrained to 1 for all branches, whereas in the alternative model, k is allowed to differ between reference and test groups. Acceptance or rejection of the alternative model is tested using a likelihood‐ratio test, but Akaike Information Criterion was also included as measures of fit of the null model and the alternative model. Four different treatments were examined on host and parasite branches for RELAX analyses which are as follows. Treatment 1: the combined parasite clade was selected as a test branch and the combined host clade was treated as a reference branch. Treatment 2: common ancestral lineage of parasite was tested against common ancestral lineage of host. Treatment 3: *I. schwarzi* as a test branch and *E. robusta* as a reference branch. Treatment 4: *I. excavata* as a test branch and *E. angophorae* as a reference branch. All treatments were also tested on concatenated mitochondrial genes. Two treatments were examined on ND‐3 gene as it wasn't found in *I. schwarzi*. No significant differences in purifying selection efficiency were found between host and parasite species in any treatments. ω reported here is calculated under the accepted null model.

## 4 DISCUSSION

Our initial aim was to examine whether the evolution processes of mitochondrial genes are faster in allodapine obligate social parasite species which exhibit much smaller effective population sizes than their hosts. Because *N*_e_ has been predicted to affect the pattern and rates of molecular evolution (Woolfit, 2009; Woolfit & Bromham, 2003), a priori we expected socially parasitic species have faster rates of molecular evolution than their hosts due to their greatly reduced effective population sizes.

ω values calculated by BUSTED analyses indicated that all host and parasite mtDNA genes have undergone purifying selection, which is the major mode of selection acting on mitochondrial genes (Castellana et al., 2011; Soares et al., 2013; Stewart et al., 2008). However, we found no evidence that the efficiency of purifying selection is reduced in parasite lineages due to possible increased genetic drift associated with much smaller *N*_e_. In other words, the RELAX outcome provided no support to our hypothesis that the rate of molecular evolution of mtDNA genes is higher in parasite species than their hosts in any of the treatments.

Our finding here is inconsistent with some previous studies, which found inverse relationships between *N*_e_ and rates of molecular evolution (Bromham & Leys, 2005; DeSalle & Templeton, 1988; Johnson & Seger, 2001; Spradling et al., 2001; Weinreich, 2001; Woolfit & Bromham, 2003; Wu & Li, 1985). This discrepancy might be partly due to some deficiencies in previous studies, but also to different approaches used for comparisons. For instance, Wu and Li (1985) showed that globin genes evolve at higher rates in rodents with smaller *N*_e_ compared to primate lineages. However, each analysis in their study was based on a single comparison, which might have limited the breadth of their conclusions. In addition, Spradling et al. (2001) found differences in rates of Cyt‐b among rodent species with different *N*_e_s, but for the rate comparison, they didn't use phylogenetically independent comparison methods (e.g., as developed by Felsenstein, 1985) which led to inflated type I and type II errors (Harvey & Rambaut, 1998). Johnson and Seger (2001) also confirmed an opposite relationship between *N*_e_ and rates of molecular evolution using island and mainland bird species; however, they used a small and taxonomically restricted dataset for their comparison. Later, Wright et al. (2009) used a much larger and more varied dataset and found no significant difference between island and mainland species.

Although Woolfit and Bromham (2003) found increased rates of 16S rRNA evolution in endosymbiotic bacteria and fungi with small effective population sizes, they found that A+T base composition of the same gene, as another indicator of increased genetic drift, is not significantly different between compared taxa. Furthermore, Bromham and Leys (2005) used hosts and parasites of bumble bees, allodapine bees and ants for their comparisons and found consistently higher rates of molecular evolution in parasite lineages than hosts. However, they used concatenated mtDNA, nuclear and ribosomal genes for almost all their comparisons, which may conflate quite different evolutionary processes (e.g., recombinant nuclear genes with non‐recombinant mt genes). In the case of the allodapine bees in their study, they did not compare inquiline species with their corresponding hosts, while in our study we explicitly compared rates between each host and its specific parasite.

Our finding in this study raises a critical question which is: why the comparisons failed to support the predicted differences in efficiency of positive and purifying selection (using RELAX method) between hosts and their social parasites? We now put forward several possibilities that might explain this outcome.

One possible interpretation is that *N*_e_ of inquilines is not reduced enough to affect the rates of molecular evolution despite their comparatively lower *N*_e_ compared to the host. For example, while it has been found that *N*_e_ of allodapine inquilines is about an order of magnitude lower than their hosts based on incidences of parasitization (Shokri Bousjein et al., 2016), it is possible that its absolute *N*_e_ is still large enough to allow effective purifying selection. In addition, *N*_e_ could also be influenced by variation in the number of offspring per individual, sex ratio (Hedrick & Parker, 1997; Shokri Bousjein et al., 2017), reproductive skew among individuals, and colony productivity. These factors are connected to each other in a complex way in allodapine bees (Schwarz et al., 2007). For instance, reproductive skew can be affected by relatedness among nest mates (Harradine et al., 2012; Langer et al., 2004), however, skew is also linked with colony productivity (Schwarz, 1986), and both of these co‐vary with sex allocation (Schwarz, 1994). Because of these complicating factors, a precise estimation of *N*_e_ is problematic. Nevertheless, genetic methods such as using the d_N_/d_S_ ratio are an attempt to obtain an accurate measure of *N*_e_; however, these methods are impractical when there have been historical changes in population size or selection on coding genes (Gregory & Witt, 2008; Whitney & Garland, 2010).

An alternative issue concerns how great the effect of any change in *N*_e_ should be on the rates of molecular evolution; so, while it is clear that *N*_e_ may potentially have considerable effects on the rates of evolution, estimation of the magnitude of that effect is not simple (Bromham & Leys, 2005; Woolfit, 2009). Previous studies (e.g., Bachtrog, 2008; Woolfit, 2009) have suggested that the distribution of fitness effects (proportion) of both slightly deleterious and advantageous mutations must be considered when the degree of the effect of a change in *N*_e_ on rates of evolution is determined. Woolfit (2009) argued that if two lineages exhibit discretely contrasting *N*_e_ (one with the large [*N*_e_L], and another with the small [*N*_e_S]), then the proportion of slightly deleterious mutations (that have a selective coefficient between 1/*N*_e_L and 1/*N*_e_S) should determine the difference in evolutionary rates between two lineages. Earlier studies obtained inconsistent results on the size of that proportion. Ohta (1977) suggested that the effect of a change in population size on the rate of evolution is expected to be quite large because a substantial proportion of mutations fall in the range from 1/*N*_e_L to 1/*N*_e_S. By contrast, Kimura (1979) suggested fewer mutations will be in this range, therefore the difference in evolutionary rates between lineages with different *N*_e_ will also be low. However, Silander et al. (2007) argued that the distribution of selective coefficients of slightly deleterious mutations is dynamic between taxa and even within a species. Initially, slightly deleterious mutations were often taken into account to determine the difference in rates of evolution because they are more common in small population, while slightly advantageous mutations are assumed to be rare in such lineages (Woolfit, 2009). However, Charlesworth and Eyre‐Walker (2007) found that slightly advantageous mutations that are under positive selection are also relatively common in small populations. This subsequently led Woolfit (2009) to suggest that the distribution of fitness effects of both advantageous and deleterious mutations should be considered while comparing rates of molecular evolution.

Another possibility is that other aspects aside from *N*_e_ might have a substantial influence on rates of evolution. Previous studies found that fecundity is strongly positively correlated with rates of evolution (Bomham & Ley 2005, Welch & Bromham, 2005). One possible explanation for this pattern is that the number of genome copies per generation scales linearly with fecundity, thereby generating more opportunities for DNA copy error/mutations in species with higher fecundity. This effect might be evident in a higher number of synonymous mutations and consequently higher rates of evolution in those species (Bomham & Leys, 2005). Thus, the impact of this feature on the rate of evolution is completely opposite to that of the *N*_e_ effect, with higher rates for host species (which have high fecundities) relative to inquilines (which have low fecundities; Shokri Bousjein et al., 2016).

The other possibility which may be worth considering is that the small sample sizes used for selection pressure comparisons might lack the ability to detect differences. Although our RELAX results are only based on two pairs of host‐parasite species in this study, we used RELAX 3 (within Hyphy v.2.5 which is the latest version) for selection intensity analyses according to S. L. Kosakovsky Pond (Primary developer of the Hyphy software, personal communication, October 2019), where the power of selection test is higher than previous versions given that only two classes of ω are estimated and selection test parameters are not over‐parametrized. This priority makes the RELAX method sharp enough to provide definitive results from the small set of test and reference branches which could be one for each. We therefore believe that by applying RELAX 3, we could somehow overcome the limitations of small sample sizes in this study. Nevertheless, if more sequence data of additional host‐parasite bee species pairs become available, this hypothesis could be tested more thoroughly.

Furthermore, in this study we targeted only mtDNA genes to examine the hypothesis and found no difference between evolutionary rates of mtDNA genes among host and parasite species in any of the treatments. It is possible that sole use of mtDNA genes might not be adequate to examine the hypothesis. This might be partly due to the fact that mitochondrial genes have been less likely involved in the adaptation process given their role in basic metabolic functions (respiration; Galtier et al., 2009). In addition, given that the mtDNA genes are maternally inherited and have reduced effective population size, they therefore do not represent the true genomic inheritance of organisms (Ballard & Whitlock, 2004). Our findings suggested that nuclear genes must be included in order to discover potential changes in selection pressure between the host and parasite species.

In conclusion, our analyses suggest that relatively small *N*_e_ of parasite lineages has seemingly no strong impact on rates of molecular evolution of their protein‐coding mtDNA genes compared to their hosts. This does not match the patterns found by Bromham and Leys (2005) who included *Exoneura* and *Inquilina* in their study and it also conflicts with some broader theoretical considerations. Our results indicate a need to consider a variety of theoretical bases for comparative rates of evolution for host/parasite relationships, and these may include the evolution of high‐stake arms races into more benign relationships such as symbiosis or mildly deleterious associations. At present, the evolution of more benign species interactions has not been explored in terms of rates of molecular evolution and we argue that our data calls for such an examination in future studies.

## CONFLICT OF INTEREST

The authors have no conflict of interest to declare.

## AUTHOR CONTRIBUTION

**Nahid Shokri Bousjein:** Conceptualization (lead); Data curation (lead); Formal analysis (equal); Funding acquisition (equal); Investigation (equal); Methodology (equal); Software (equal); Validation (equal); Visualization (lead); Writing – original draft (lead); Writing – review & editing (equal). **Simon Tierney:** Conceptualization (equal); Formal analysis (equal); Investigation (equal); Methodology (equal); Software (equal); Supervision (equal); Writing – review & editing (supporting). **Michael Gardner:** Conceptualization (equal); Funding acquisition (equal); Resources (equal); Supervision (equal); Validation (equal); Writing – review & editing (supporting). **Michael Schwarz:** Conceptualization (equal); Funding acquisition (equal); Investigation (equal); Resources (equal); Supervision (lead); Validation (equal); Visualization (equal); Writing – review & editing (equal).

## Supporting information

Supplementary Material

## Data Availability

All mtDNA gene sequences were deposited in GenBank (See accession numbers in Table 1).
