# Supplementary material for: Does effective population size affect rates of molecular evolution: Mitochondrial data for host/parasite species pairs in bees suggests not
Source: Ecol Evol. 2022 Feb 7;12(2):e8562. doi: 10.1002/ece3.8562 (PMC8820120; doi:10.1002/ece3.8562)
Supplement: Supplementary file 1 — Supplementary Material [file ECE3-12-e8562-s001.docx]

**SUPPLEMENTARY MATERIAL**

**Does effective population size affect rates of molecular evolution: mitochondrial data for host/parasite species pairs in bees suggests not**

Nahid Shokri Bousjein, Simon M. Tierney, Michael G. Gardner, and Michael P. Schwarz

**Table Legend**

**Table S1**. Accession numbers of complete mitochondrial genome sequence of reference species used in this study.

**Table S2**. Transcriptome read and assembly summary.

**Table S3**. mtDNA sequence coverage depth.

**Figure Legend**

**Figure S1**. Bayesian phylogenetic trees based on (A) ATP6, (B) ATP8, (C) CO1, (D) CO2, (E) CO3, (F) Cytb, (G) ND-1, (H) ND-3, (I) ND-4, (J) ND-4L and (K) ND-5 mtDNA genes, with posterior probability node support. Parasites and hosts are indicated by coloured branches: pink for hosts and blue for parasites. The lines to the right of the terminal branches link each host to its associated parasite. For trees B and J *Hylaeus dilatatus* is included as outgroups and for the rest of trees *Hylaeus dilatatus* and *Colletes gigas* are included as outgroups.

**Figure S2**. Treatments used to test for relaxed selection on portion of the inferred concatenated Bayesian tree (containing two focal host and their respective parasite species). Test and reference branches are indicated by orange and green colours respectively for each treatment. Host and parasite species are shown in pink and blue fonts respectively.

Table **S**1. Accession numbers of complete mitochondrial genome sequence of reference species used in this study. Full reference citations are included below.

| **Taxon name** | **Family** | **Common name** | **Accession** | **Reference** |
| --- | --- | --- | --- | --- |
| ***Apis mellifera scutellata*** | **Apidea** | **African honeybee** | **KJ601784** | **Gibson and Hunt, 2014** |
| ***Apis florae*** | **Apidea** | **Red dwarf honeybee** | **JX982136** | **Wang et al. 2013** |
| ***Melipona bicolor*** | **Apidea** | **Guaraipo bee** | **NC_004529** | **Silvestre et al. 2008** |
| ***Melipona scutellaris*** | **Apidea** | **Urussu bee** | **NC_026198** | **Pereira et al. 2016** |
| ***Bombus ignitus*** | **Apidea** | **Bumble bee** | **NC_010967** | **Cha et al. 2007** |
| ***Colletes gigas*** | **Colletidae** | **Plasterer bee** | **NC_026218** | **Huang et al. 2016** |
| ***Hylaeus dilatatus*** | **Colletidae** | **Chalk yellow face bee** | **KP126800** | **Tan et al. 2016** |

References (pertaining to Table S1)

Cha, S. Y., H. J. Yoon, E. M. Lee, M. H. Yoon, J. S. Hwang, B. R. Jin, Y. S. Han, and I. Kim. 2007. The complete nucleotide sequence and gene organization of the mitochondrial genome of the bumblebee, *Bombus ignitus* (Hymenoptera: Apidae). Gene 392:206-220. http://doi: [10.1016/j.gene.2006.12.031](https://doi.org/10.1016/j.gene.2006.12.031).

Gibson, J. D., and G. J. Hunt. 2014. The complete mitochondrial genome of the invasive Africanized Honey

Bee, *Apis mellifera scutellata* (Insecta: Hymenoptera: Apidae. Mitochondrial DNA 27:561-562. http://doi: 10.3109/19401736.2014.905858.

Huang, D., T. Su, L. Qu, Y. Wu, P. Gu, B. He, X. Xu, and C. Zhu. 2016. The complete mitochondrial genome of the *Colletes gigas*

(Hymenoptera: Colletidae: Colletinae). Mitochondrial DNA 27:3878-3879. http://doi: [10.3109/19401736.2014.987243](https://doi.org/10.3109/19401736.2014.987243).

Pereira, U. P., A. M. Bonetti, L. R. Goulart, A. R. Santos, G. C. Oliveira, S. Cuadros, and C. Ueira-Vieira. 2016. Complete mitochondrial genome sequence of *Melipona scutellaris*, a Brazilian stingless bee. [Mitochondrial DNA](https://www.tandfonline.com/imdn21) 27:3387-3388. http://doi: [10.3109/19401736.2015.1018233](https://doi.org/10.3109/19401736.2015.1018233).

Silvestre, D., M. Dowton, and M. C. Arias. 2008. The mitochondrial genome of the stingless bee *Melipona bicolor* (Hymenoptera, Apidae, Meliponini): sequence, gene organization and a unique tRNA translocation event conserved across the tribe Meliponini. Genet. Mol. Biol. 31:451-460. http://doi: 10.1590/S1415-47572008000300010.

Tan, M., R. Zhang, C. Hardman, and X. Zhou. 2016. Mitochondrial genome of *Hylaeus dilatatus* (Hymenoptera: Colletidae). Mitochondrial DNA 27:3975-3976. http://doi: [10.3109/19401736.2014.989511](https://doi.org/10.3109/19401736.2014.989511).

Wang, A. R., M. J. Kim, J. S. Park, Y. S. Choi, R. Thapa, K. Y. Lee, and I. Kim. 2013. Complete mitochondrial genome of the dwarf honeybee, *Apis florea* (Hymenoptera: Apidae). Mitochondrial DNA 24:208-210. http://doi:[10.3109/19401736.2012.744986](https://doi.org/10.3109/19401736.2012.744986).

Table S2. Transcriptome read and assembly summary.

| **Taxa** | **Relation** | **body segment** | **QA paired-end reads (read length)** | **Total sequences after trimming** | ***De novo* assembled transcripts (contig N50)** | **Total aligned reads (% proper pairs)** |
| --- | --- | --- | --- | --- | --- | --- |
| ***E. robusta*** | **Host** | **Head** | **29,320,666 (100bp)** | **20985442** | **82102 (968bp)** | **37226484 (52.83%)** |
|  |  | **Metasoma** | **27,303,651 (100bp)** | **16655057** | **28372 (552bp)** | **30498594 (55.68%)** |
| ***I. schwarzi*** | **Parasite** | **Head** | **30,839,546 (100bp)** | **22396119** | **65944 (992bp)** | **40886113 (60.78%)** |
|  |  | **Metasoma** | **32,631,666 (100bp)** | **23734470** | **77700 (1189bp)** | **43935616 (51.03%)** |
| ***E. angophorae*** | **Host** | **Head** | **35,318,489 (100bp)** | **25741159** | **66426 (1080bp)** | **47616902 (77.62%)** |
|  |  | **Metasoma** | **34,386,652 (100bp)** | **24592950** | **76988 (1135bp)** | **44565173 (61.88%)** |
| ***I. excavata*** | **parasite** | **Head** | **32.160,656 (100bp)** | **24967610** | **77109 (1526bp)** | **46464257 (73.44%)** |
|  |  | **Metasoma** | **37,715,844 (100bp)** | **29259350** | **69007 (1301bp)** | **54356494 (65.36%)** |

Table S3. mtDNA sequence coverage depth. The first row indicates mean sequence coverage depth per nucleotide base (± s.e.) for transcripts of mtDNA orthologous genes. The maximum depth for a single nucleotide base within each transcript is presented in square brackets and the sample size (*n*) represents the full length of the unedited transcript (number of bases). Data not available for three mtDNA genes of *I. schwarzi.*

| **mtDNA**  **genes**  **Species** | **ATP6** | **ATP8** | **CO1** | **CO2** | **CO3** | **Cyt-b** |
| --- | --- | --- | --- | --- | --- | --- |
| ***E. robusta*** | **40845.29±**  **51903.94** | **40845.29±**  **51903.94** | **45725.88±**  **24890.49** | **40845.29±**  **51903.94** | **34627.03±**  **46309.80** | **10981.58±**  **7903.98** |
|  | **[245927]** | **[245927]** | **[101606]** | **[245927]** | **[245927]** | **[32668]** |
|  | **n = 3754** | **n = 3754** | **n = 2852** | **n = 3754** | **n = 5186** | **n =2356** |
| ***E. angophorae*** | **16900.99±**  **18738.06** | **16900.99±**  **18738.06** | **36389.30±**  **23593.51** | **16900.99±**  **18738.06** | **15557.903±**  **17675.71** | **7241.53±**  **6729.82** |
|  | **[83137]** | **[83137]** | **[109632]** | **[83137]** | **[83137]** | **[27454]** |
|  | **n = 2339** | **n = 2339** | **n = 2848** | **n = 2339** | **n = 4671** | **n = 2386** |
| ***I. schwarzi*** | **20558.93±39881.38** | **20558.93±39881.38** | **43874.29±33564.59** | **22297.61±36449.83** | **18678.74±36015.61** | **-----** |
|  | **[200586]** | **[200586]** | **[157338]** | **[200586]** | **[200586]** | **-----** |
|  | **n = 3077** | **n = 3077** | **n = 3067** | **n = 3845** | **n = 3862** | **-----** |
| ***I. excavata*** | **7912.18±12095.31** | **7912.18±12095.31** | **21023.45±17168.40** | **11378.52± 18462.48** | **7587.12±10680.48** | **2848.26±2791.28** |
|  | **[65897]** | **[65897]** | **[73903]** | **[98885]** | **[65897]** | **[11284]** |
|  | **n =2706** | **n =2706** | **n = 3223** | **n = 3017** | **n = 3659** | **n = 3090** |

Table S3. Continued.

| **mtDNA**  **genes**  **Species** | **ND-1** | **ND-3** | **ND-4** | **ND-4L** | | **ND-5** |
| --- | --- | --- | --- | --- | --- | --- |
| ***E. robusta*** | **1114.69±**  **797.81** | **2692.59±**  **5118.60** | **1357.3±**  **1418.59** | **1231.52±**  **1393.71** | | **1047.35±**  **1386.47** |
|  | **[2835]** | **[22143]** | **[5719]** | **[5719]** | | **[5719]** |
|  | **n =1182** | **n =1191** | **n = 2841** | **n =3169** | | **n =3579** |
| ***E. angophorae*** | **253.354±**  **142.80** | **7.539±**  **5.88** | **650.97±**  **614.25** | | **650.97±**  **614.25** | **105.91±**  **92.33** |
|  | **[678]** | **[19]** | **[2285]** | | **[2285]** | **[384]** |
|  | **n = 2037** | **n = 623** | **n = 2743** | | **n = 2743** | **n = 1852** |
| ***I. schwarzi*** | **-----** | **-----** | **2114.44±2197.66** | | **2114.44±2197.66** | **509.50±712313** |
|  | **-----** | **-----** | **[8443]** | | **[8443]** | **[2893]** |
|  | **-----** | **-----** | **n = 1732** | | **n = 1732** | **n = 1522** |
| ***I. excavata*** | **1465.23±1813.33** | **33.18±21.09** | **695.48±720.44** | | **695.487±720.44** | **283.20±337.22** |
|  | **[6472]** | **[77]** | **[2735]** | **[2735]** | | **[1526]** |
|  | **n = 2153** | **n = 613** | **n = 3052** | **n = 3052** | | **n = 2107** |


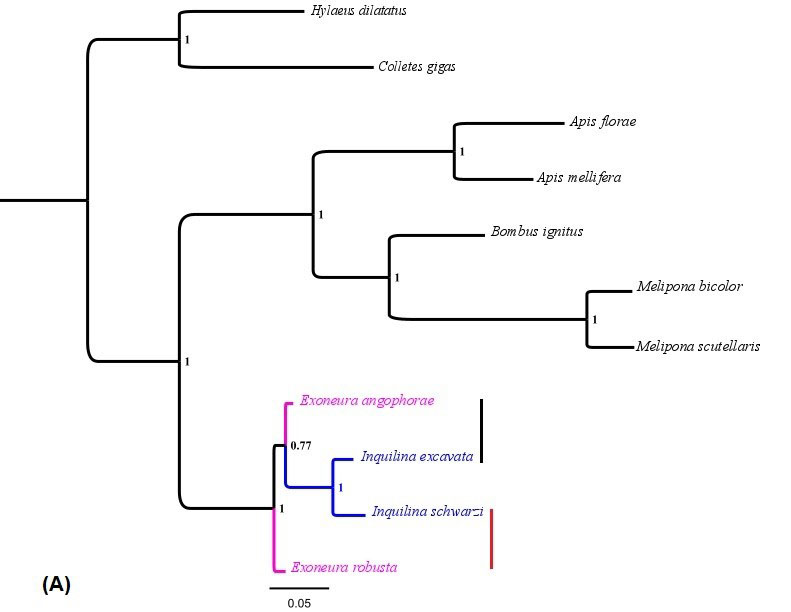

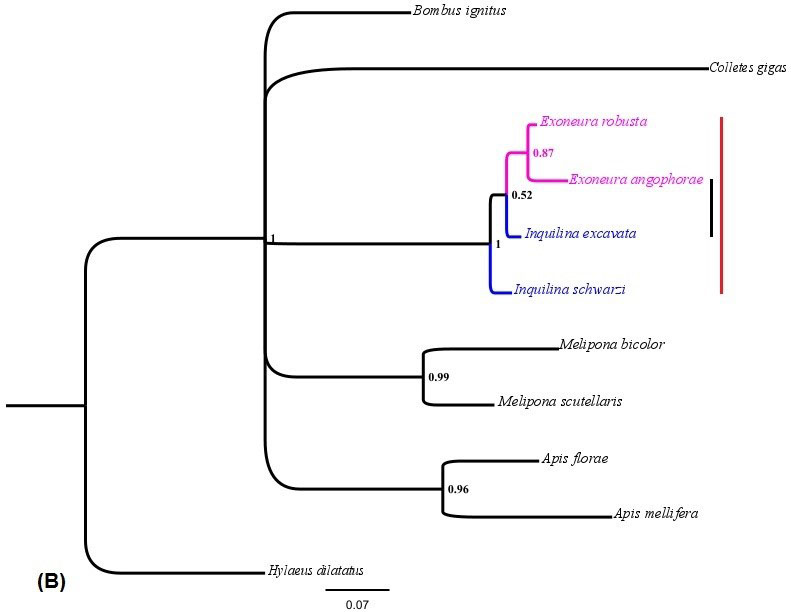


**Figure S1.**


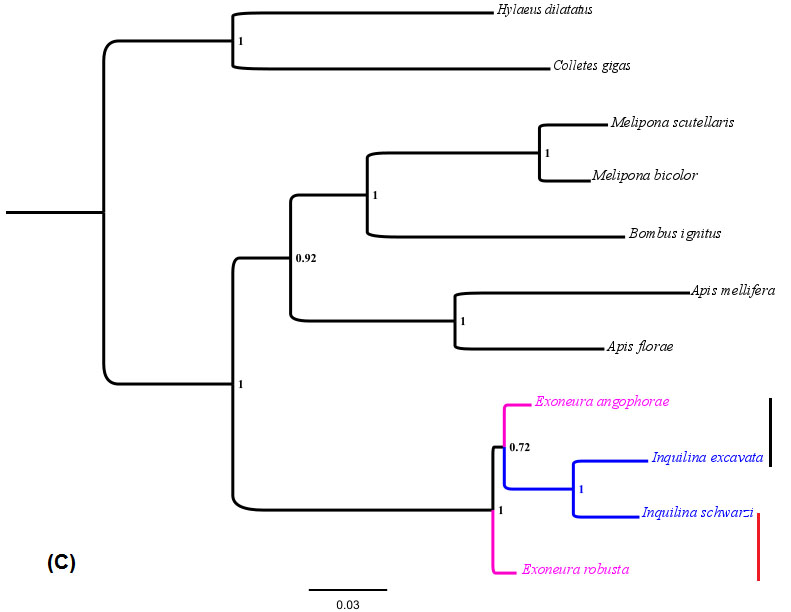

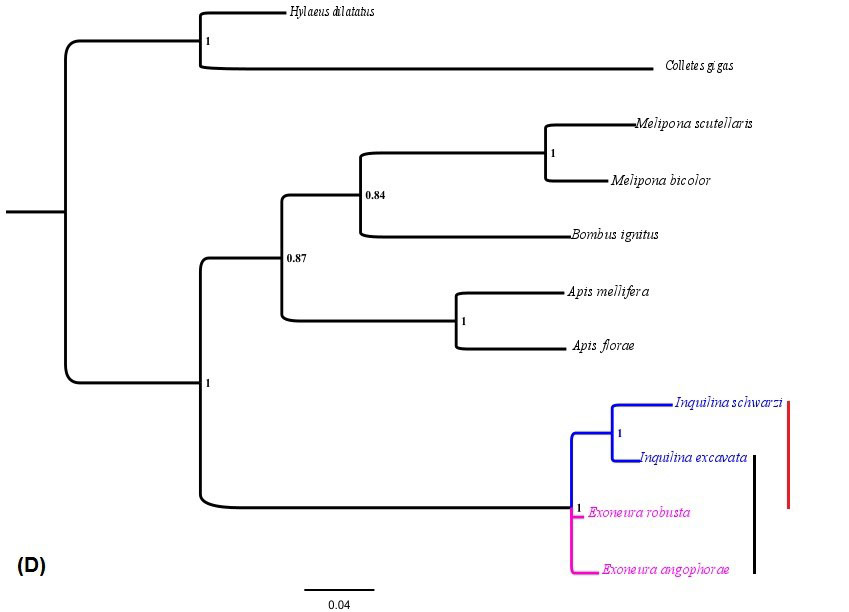


**Figure S1. Continued.**

**
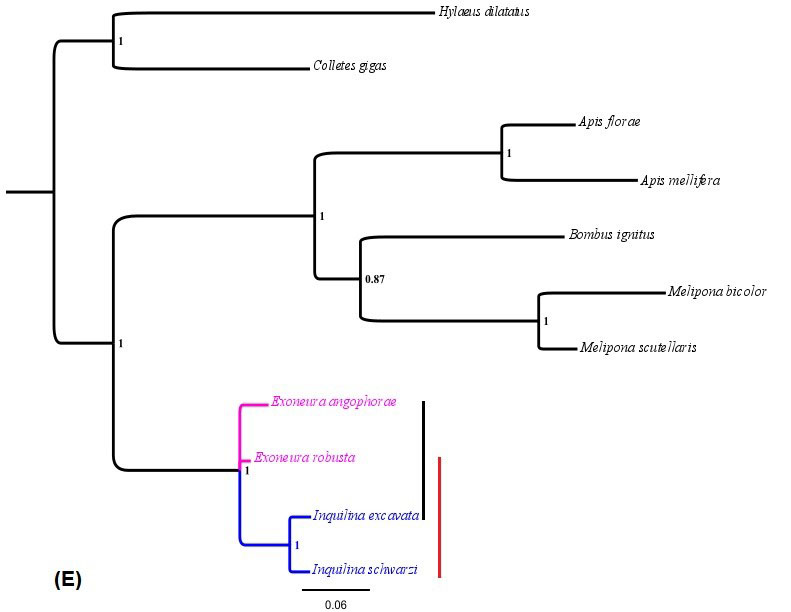

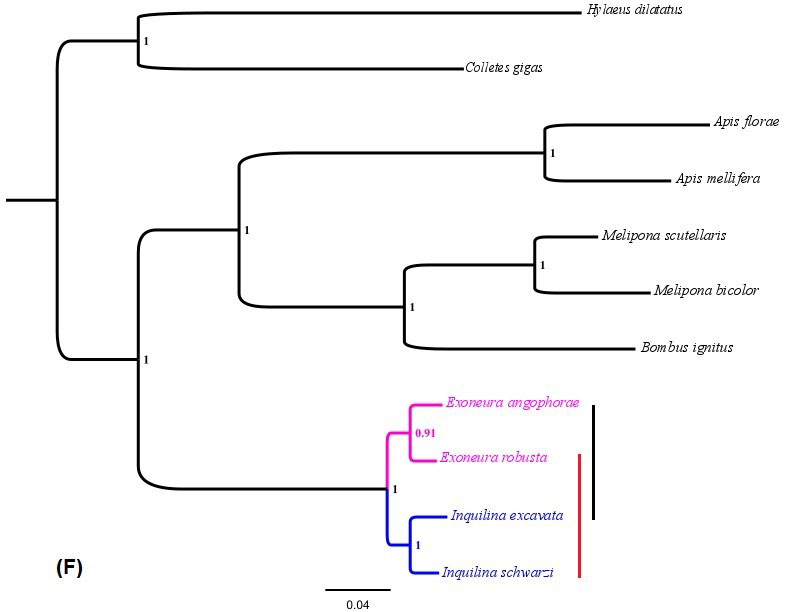
**

**Figure S1. Continued.**


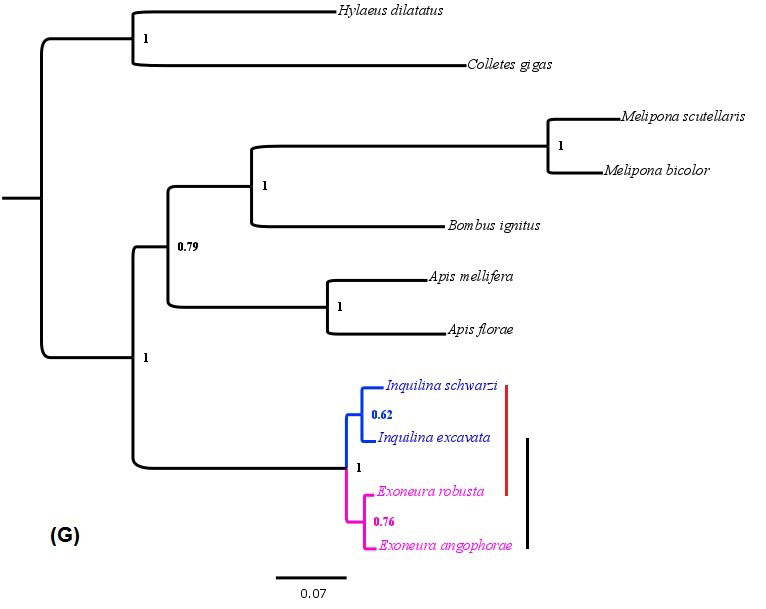

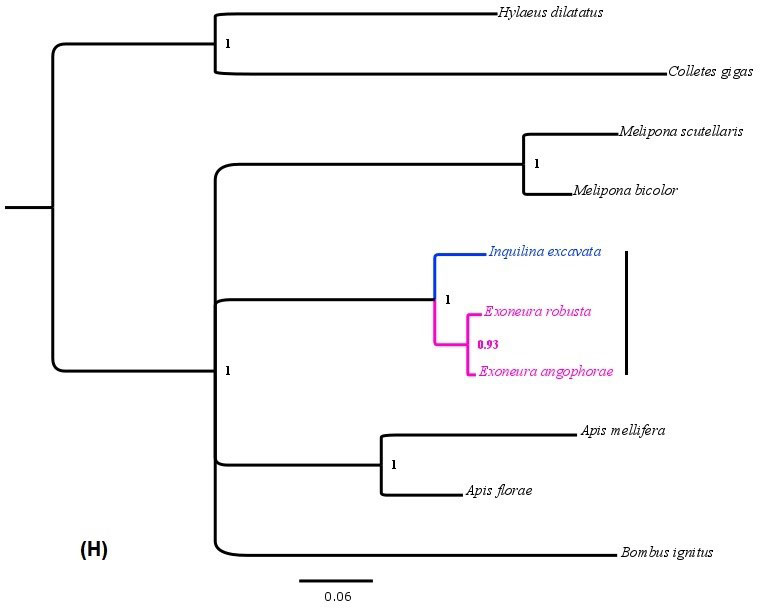


**Figure S1. Continued.**


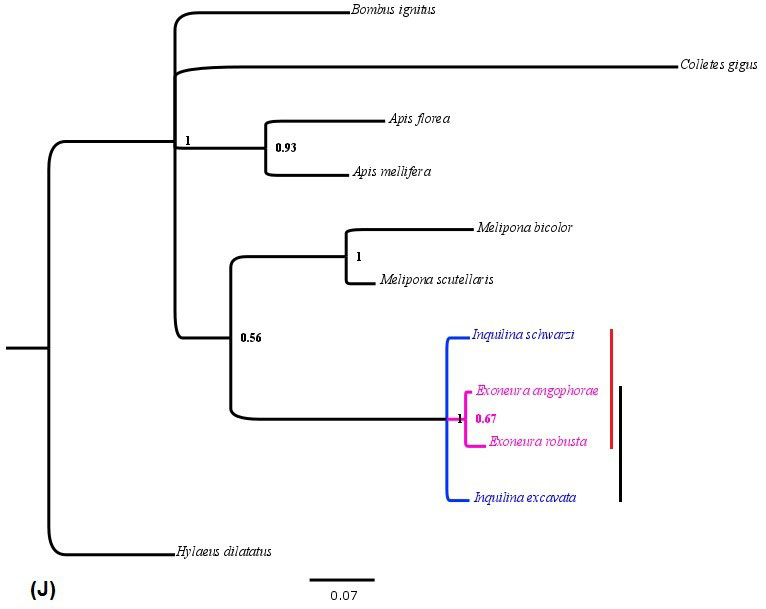

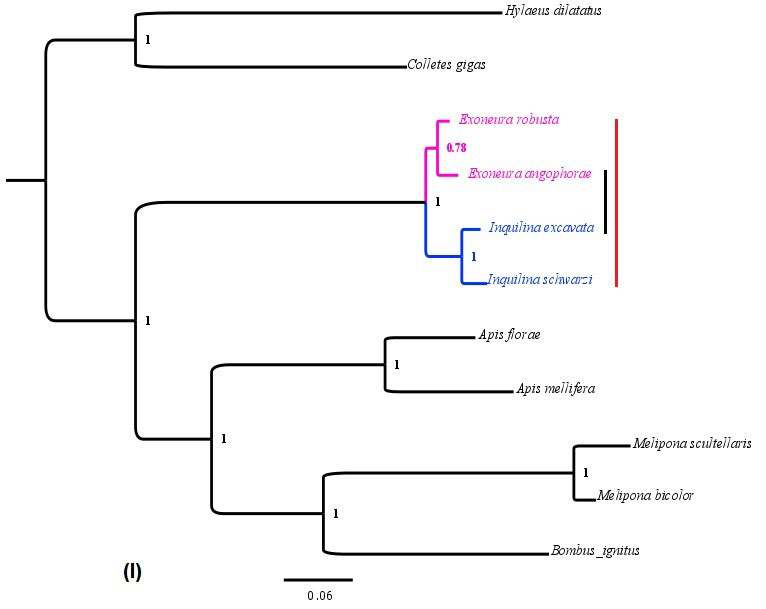


**Figure S1. Continued.**


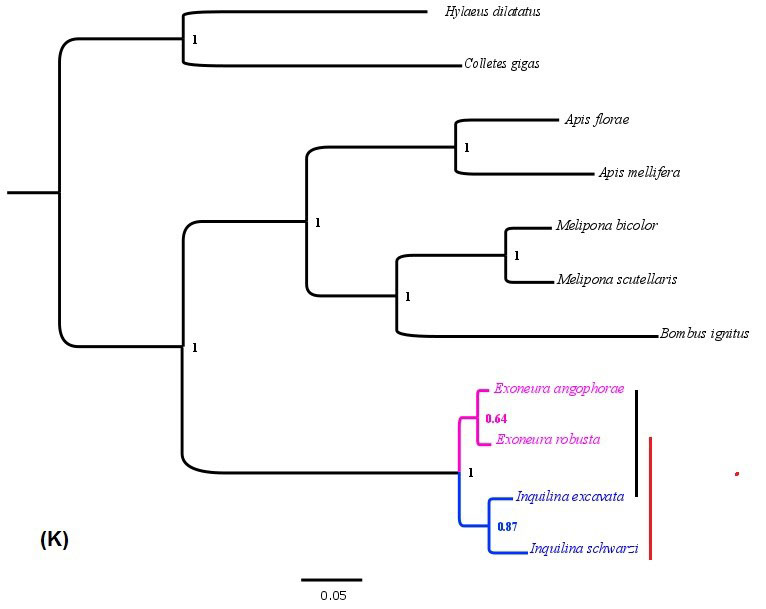


**Figure S1. Continued.**


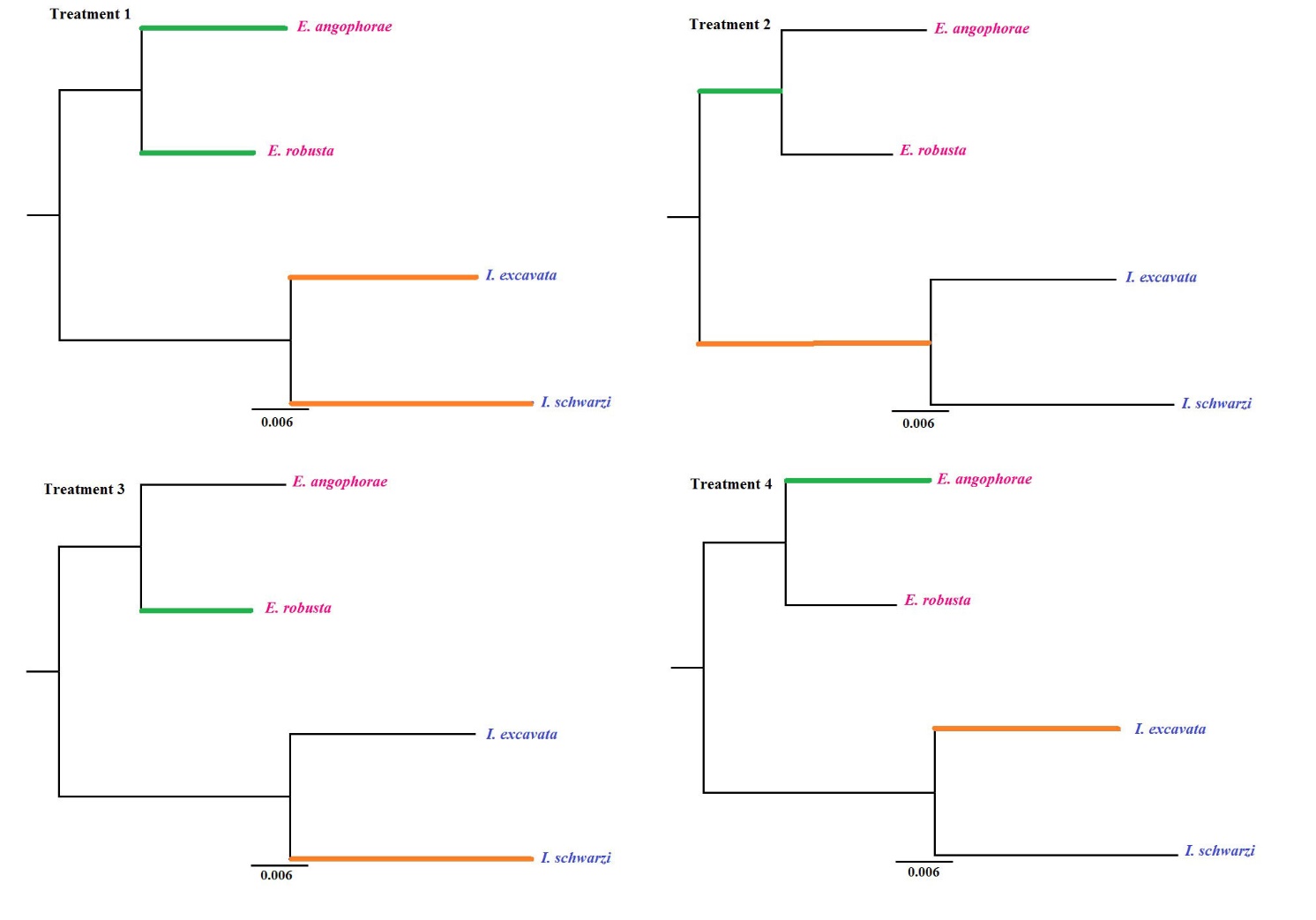


**Figure S2.**
